# Impact of product subsidies on R&D investment for new energy vehicle firms: Considering quality preference of the early adopter group

**DOI:** 10.1371/journal.pone.0236626

**Published:** 2020-07-31

**Authors:** Weidong Meng, Ye Wang, Yuyu Li, Bo Huang

**Affiliations:** 1 School of Economics and Business Administration, Chongqing University, Chongqing, China; 2 School of Economics and Management, Chongqing Normal University, Chongqing, China; Shandong University of Science and Technology, CHINA

## Abstract

Different consumer groups accept new energy vehicles sequentially from the perspective of innovation diffusion theory, and the early adopter group has recently been identified. By assuming that the density of early adopters is increasing at minimum acceptable quality thresholds, this paper proposes a vertical quality differentiation model of product R&D with product subsidies. The impact of product subsidies on the R&D investment of new energy vehicle firms is discussed. We show that the early adopters’ characteristics may affect the stagnant marginal R&D investment of new energy vehicle firms by increasing sales, which determines the impact mechanism of product subsidies. For firms with decreasing marginal R&D investments, insufficient R&D investments result from financial constraints. If insufficient R&D resources deter firms from conducting R&D, substantial unit subsidies invariably incentivize firms to spend their entire R&D budget. Firms with increasing marginal R&D investments, insufficient R&D profits, or financial constraints are prevented from increasing R&D investment. Product subsidies generally have a crowding-in effect on firms not subject to financial constraints, and this effect increases with the unit subsidy. However, the existence of a crowding-in effect may require sufficiently large unit subsidies. In both situations, product subsidies cannot modulate financial constraints if the firm has spent its entire R&D budget. In the first situation, we also show that product subsidies should be replaced by a funding support policy. In contrast, the second situation shows that a funding support policy should be coordinated with product subsidies.

## Introduction

Recently, new energy vehicle (NEV) development has captured the interest of both the public and private sectors, which is of great significance for the sustainable development of energy and the environment [1–3]. Though it seems unrealistic for NEVs to replace internal combustion engine vehicles (ICEVs) now because their technologies (quality) still need considerable improvement [4, 5]. To encourage strengthening R&D, some governments have provided strong R&D subsidy policies for NEV firms [6–10]. China, for example, has spent more than 1000 billion Chinese yuan up to now on a product subsidy policy for firms from the central government [11–14]. The policy did not achieve the government’s expected targets [9, 15]. Therefore, this paper aims to provide some useful suggestions for this timely and critical issue, on how to encourage NEV firms to increase their R&D investment by product subsidies.

The government’s considerations in funding incentives for private R&D is usually guided by ancillary benefits, leading to underfunded R&D investment in private firms [16, 17]. However, the conclusions reached by a large number of similar studies do not indicate the expected effects of subsidies on the R&D investment of firms [18–20]. These inconsistencies are easily identifiable, among others a crowding-in effect, a crowding-out effect, no significant effect, and mixed effect [21–24]. A fundamental reason for inconsistencies in the policies guiding the granting of subsidies is that they inevitably are not only specific to the economic environment, but also applicable to distinct channels, and influenced by numerous factors [24, 25].

Seminal in this field is to investigate which aspects are effective in the granting of subsidies. Hassine and Mathieu [26] found that government subsidies create a significant leverage effect, and firms inside the industry clusters receive more R&D subsidies and invest more R&D funds comparing with firms outside clusters. Bai et al. [27] found that R&D subsidies increase the green innovation of energy-intensive firms, where the impact is more robust for state-owned firms and SMEs. Yu et al. [28] found that the relationship between the effect of government subsidies and renewable energy firms’ R&D intention presents an inverted-U shape, which is further moderated by the attributes of firm ownership. Yang and Xiao [29] considered that the government provides subsidies for manufacturers with a green level floor and, no matter how high this floor is, retailers as leaders in the supply chain will create higher R&D investment than manufacturers as leaders. Howell [30] found that an early-stage R&D subsidies positive impact on the firm’s innovation, though these effects are more forceful for firms with financial constraints.

In this field of research, there is a lack of those that study NEV firms by focusing on how they are uniquely affected by subsidies. The dearth of studies we examine now may be attributed to insufficient data, or the classification of NEV firms as automotive, manufacturing, or environmental R&D [31–33]. Jiang et al. [31] found that government subsidies will increase the NEV firms’ R&D investment, which is significant for assembly firms but not for supporting firms. Xiong, Fan, and Liu [34] found that appropriate fiscal subsidies have an incentive effect for manufacturers’ R&D investment, and too many subsidies will have a negative impact. Strategic decision-making elements such as the executive shareholding ratio, ownership concentration, and proportion of independent directors have a significant impact on the incentive effect of fiscal subsidies in R&D investment. Liu and Zhao [35] found that a government’s subsidy policy can effectively promote the technological progress of NEV firms. However, market end subsidies shall be dropped out timely while the technology end subsidies should be strengthened.

**Fig 1:**
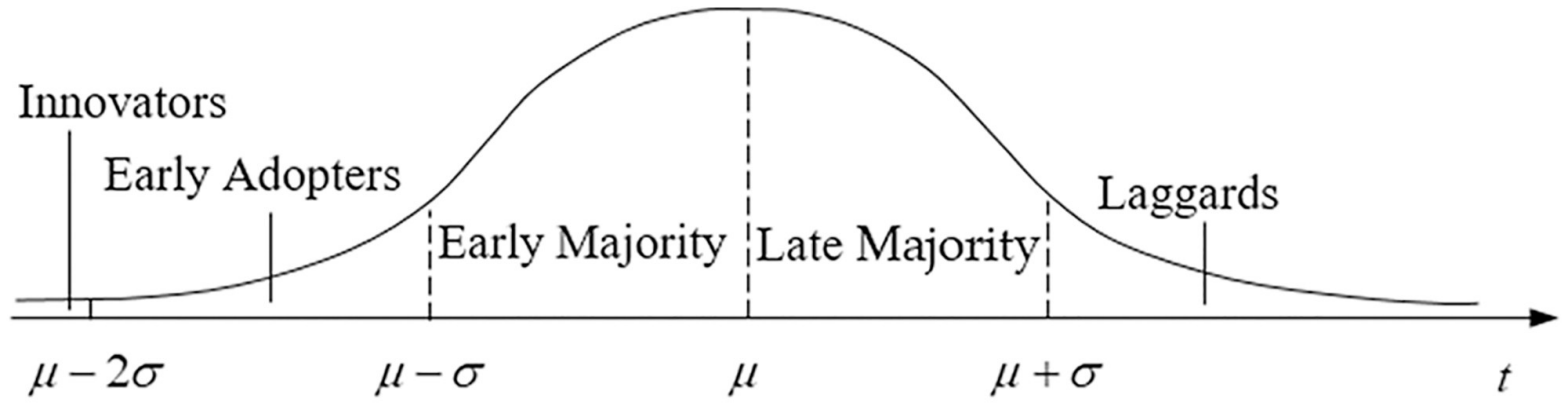
Categorizing adopters according to a new product life cycle.

Two aspects should be of concern in the NEV market, namely the phenomenon of consumer grouping, and an existing acceptable quality threshold for early adopters. These aspects also show characteristic features. While NEVs are new, environmentally friendly products, vehicles in themselves are a longstanding means of human transportation. Rogers [36] posits that the five categories of early adopters of green NEVs follow a temporal sequence in adopting innovation, as illustrated in Fig 1. Furthermore, many scholars recognized that the current consumer group is the early adopters in related research [37–40].

Furthermore, these aforementioned researchers also established that the main concerns of early adopters are price, environmental friendliness and energy efficiency, and the quality of NEVs [37–40]. The quality primarily refers to NEV performance specifications, such as battery range and charging. A number of researchers affirm that discounts, or environmental friendliness and energy efficiency are the most prominent factors contributing to the growth of the NEVs market [39, 40]. However, these findings are negated by the development of China’s NEV market. Despite subsidized NEV products, their price being competitively with ICEVs, their market share currently accounts for less than 3% of the entire vehicle market [41]. In contrast, the concerns of consumer about NEV quality, such as “range anxiety,” “charging anxiety,” imply that the minimum acceptable for quality threshold for early adopters is subjective, especially when comparing ICEV performances.

Ongoing studies into the purchase decision-making process of NEV consumers supports this view [42–47]. Hackbarth and Madlener [48] found that the quality of alternative fuel vehicles has to meet some minimum requirements for consumers who are willing to pay considerable amounts for the improvement of alternative fuel vehicles on driving range, fuel availability, and recharging time. Neaimeh et al. [49] found that if the distance of a journey is above the single-charge range of the battery electric vehicle, taking several hours to charge with standard chargers is unacceptable for consumers. Noel et al. [50] found that although electric vehicles have greatly improved in recent years, common barriers like range and charging infrastructure continue to persist.

What distinguishes this study is that we consider group of early adopters who have a high minimum threshold for acceptable quality. In general, R&D has had a peripheral effect on the improvement of quality, whereas NEV firms have marginally increased their R&D investment by increasing sales. Due consideration of the characteristics of early adopters could ostensibly lead to arresting the growing trend of marginal R&D investment, and conceivably a reversal. In response, we analyzed the product subsidy mechanism and its impact on the NEV firm’s R&D investment by comparing the fluctuations in its marginal R&D investment. Our findings indicate that product subsidy has a crowding-in effect on NEV firm that faces insufficient profits but have limited impact on inadequate R&D investment due to financial constraints. In particular, where the NEV firm has a decreasing marginal R&D investment and does not conduct R&D as limited R&D funds, product subsidy can reduce the profitable R&D investment threshold. That is, the product subsidy may indirectly impact the NEV firm’s financial constraints and result in a crowding-in effect on its R&D investment. Based on these findings, governments need to adopt a rational policy in deploying and optimizing product subsidies.

The remainder of the paper is organized as follows. In section 2, we describe the vertical differentiation model of product R&D for an NEV firm with product subsidies. The optimal R&D investment strategies and the crowding-in effect of product subsidies are obtained in sections 3 and 4. The numerical analysis is carried out in section 5, and the conclusions are given in section 6.

## The model

This study investigates one type of NEV and ICEV on the market, provided by one NEV firm and several ICEV firms, respectively. Whereas all consumers are regarded as potential buyers of ICEVs, a selected few are regarded as “the early adopters” of NEVs [36]. Our discussion below focuses on those early adopters.

The early adopters display acceptable quality threshold range $\left[\underline{x},\bar{x}\right]$, uniformly distributed, and where $\bar{x}$ is the quality of ICEVs. They distribute across this interval with increasing density *g*(*x*)*dx*. We assume, that if the energy-efficiency and environmental friendliness of the NEV exceeds their acceptable quality threshold, the consumer will choose to buy an NEV rather than an ICEV. Similarly, where ICEVs are of higher quality than NEVs, buyers are reluctant to pay more for an NEV. Moreover, when it is assumed that the ICEV market is competitively at priced at *p*, NEV prices, irrespectively, should not exceed this.

Further, consider how NEV consumers make purchase decisions. Referring to a version of the quality differentiation model [51–53], assume a consumer either buys an NEV or does not buy any. Then, under the identical budget constraint of NEV consumers *p*^*NEW*^ + *r* ≤ *y*, give the early adopter’s utility function is:

$$U\left(x,r;x^{n}\right)=r+\theta x\;if\;x\geq x^{n}\;or\;U\left(x,r;x^{n}\right)=r\;if\;x<x^{n},$$ (1)

where *x*_*n*_ is the acceptable quality threshold of consumer *n*, *p*^*NEW*^ is the NEV price, *r* is a numeraire good, *y* is the consumer income, *θ* is the price coefficient of unit quality, and $\theta \bar{x}\leq p$. From (1) and its derivative *U*_*x*_ = *θ* (the subscripts here and below denote a derivation except those in numerical form), it is clear that a consumer with utility maximization is always willing to pay for additional quality improvement if only the NEV reaches the threshold. Moreover, with a quality *x* and a price below *p*, the potential consumer number who will buy an NEV is $a\left(x\right)=\int_{\underline{x}}^{x}g\left(x\right)dx$, which satisfies *a*_*x*_ > 0 and *a*_*xx*_ > 0. This implies that the closer an NEV is to ICEV quality, in driving range and recharge time for example, the number of potential consumers who can accept them increases faster. It is worth noting that we use a continuous function *a*(*x*) instead of a discrete one for simplicity, but this does not affect our analysis and results.

In addition to the characteristics of consumers, the characteristics of the NEV firm should also be recognized. The firm has limited R&D funding *i*^*max*^, which is far from enough to bring NEV quality to $\bar{x}$. It also has a technology reserve to improve the NEV quality to *x*_0_, which satisfies $x_{0}>\underline{x}$. Moreover, with different levels of R&D investment *i*, the firm can make NEVs reach different levels of quality *x*, which satisfies *x* = *x*(*i*), *x* ≥ *x*(0) = *x*_0_, *x*_*i*_ > 0 and *x*_*ii*_ < 0.

Then, consider a three-stage decision-making process. In the first stage, the government sets the standard of product subsidies, which is the subsidy amount the firm will receive for each NEV sold. In the second stage, the firm makes R&D or R&D investment decisions. In the third stage, the firm makes NEV pricing decisions when the ICEV is priced at *p*. In fact, the NEV pricing issue does not require complicated analysis later, as the firm will always price NEVs at *p*. The reason is simple; if only the NEV quality reaches a consumer’s threshold, he or she has a reserved price *p*. Therefore, only if the firm prices NEVs at *p*, it can maximize its profits. Moreover, since the fixed marginal production cost will not affect our result, it will be simplified to zero below.

Accordingly, we get the firm’s demand function as:

$$q\left(i\right)=a\left[x\left(i\right)\right]=\int_{\underline{x}}^{x}g\left(x\right)dx,$$ (2)

where $q_{0}=q\left(0\right)=a\left(x_{0}\right)|{}_{i=0}=\int_{\underline{x}}^{x_{0}}g\left(x\right)dx>0$, and the profit function as:

π(i)=(p+s)q(i)−i, (3)

where *π*_0_ = *π*(0) = *q*_0_(*p* + *s*), (*p* + *s*)*q*(*i*) and $\bar{p}=p+s$ are the firm’s revenue and revenue per NEV.

### Optimal R&D investment strategies

In this section, we will solve the optimal R&D investment strategies of the NEV firm with product subsidy. It is worth noting that a graphical method is adopted for solving the strategy, which allows us to explain our findings more realistically with the concept of marginal R&D investment with sales. Before solving, we will analyze the change in the firm’s marginal R&D investment with the growth of its sales.

**Lemma 1.** When *x*_*i*_ > 0, *x*_*ii*_ < 0, *a*_*x*_ > 0 and *a*_*xx*_ > 0, the NEV firm’s marginal R&D investment with sales may be decreasing *i*_*qq*_ < 0 or increasing *i*_*qq*_ > 0.

**Proof of Lemma 1.** As *a*_*x*_ > 0 and *x*_*i*_ > 0, we can get *a_i_* = *a*_*x*_*x*_*i*_ < 0. And as *a*_*xx*_ > 0 and *x*_*ii*_ < 0, we can get that $a_{ii}=a_{xx}x_{i}^{2}+a_{x}x_{ii}$ can be positive or negative. From (2), we know that *q*_*ii*_ = *a*_*ii*_ can be negative or positive.

Therefore, according to the derivative rule of inverse functions, we know that *i*_*q*_ = (*q_i_*)^−1^ < 0 and *i*_*qq*_ has the opposite sign with *q*_*ii*_.

Q.E.D.

In general, the R&D of the NEV firm has a marginal diminishing effect on quality improvement. If consumers grow evenly as NEV quality improves, the firm will have an increasing marginal R&D investment with sales. However, when considering the early adopter group’s consumers have a rising density on acceptable quality thresholds, increasing pressure on the firm’s marginal R&D investment will be eased. If the early adopters’ distribution on acceptable quality thresholds has a significant enough impact, the firm has a decreasing marginal R&D investment instead; otherwise, it still has an increasing marginal R&D investment.

First, we solve the NEV firm’s optimal R&D investment strategies when its marginal R&D investment is decreasing, or *i*_*qq*_ < 0. Let *i*_1_ be the R&D investment, which makes the firm’s profits equal to *π*_0_, and we can get proposition 1 as follows.

**Proposition 1.** In the case of *i*_*qq*_ < 0:

(a) When the initial value of marginal R&D investment is no higher than the marginal revenue, and when the initial value of marginal R&D investment is higher than the marginal revenue and the firm has enough R&D funds, the optimal R&D investment strategy is the entire R&D funds;
(b) Otherwise, the optimal R&D investment strategy is zero.

**Fig 2:**
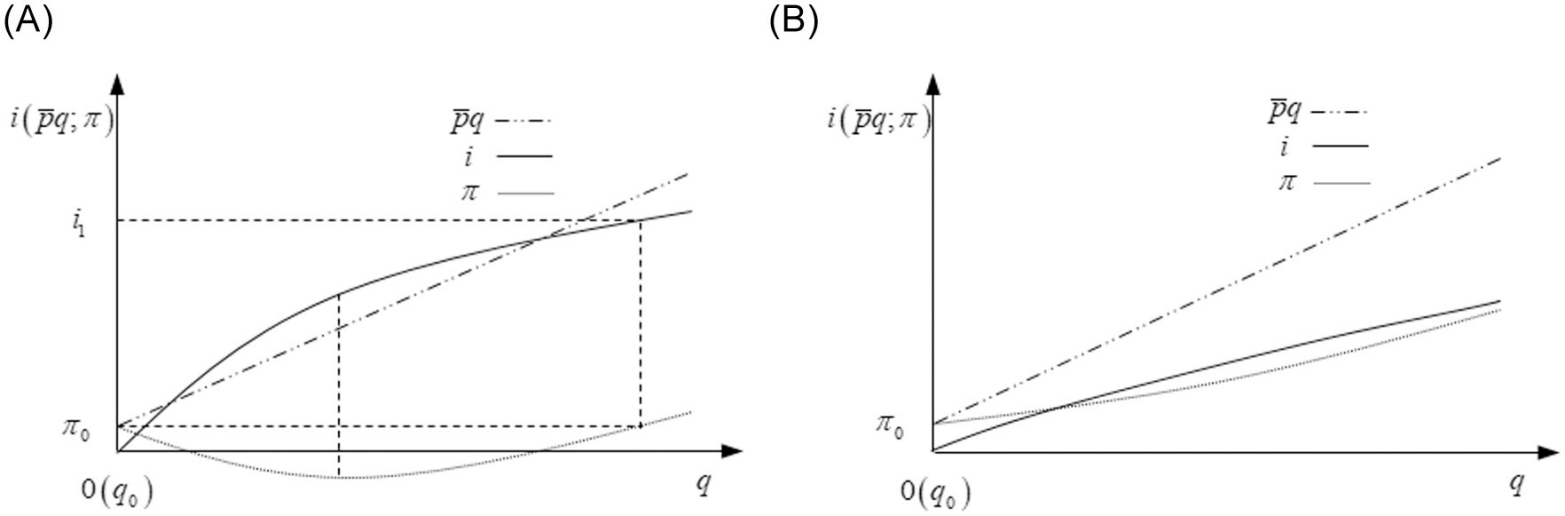
A. Changes in profits and revenue when *i*_*qq*_ < 0 and $i_{q}|{}_{i=0}>\bar{p}$. B. Changes in profits and revenue when *i*_*qq*_ < 0 and $i_{q}|{}_{i=0}\leq \bar{p}$.

**Proof of Proposition 1.** From Fig 2A and 2B, we know that the revenue curve has a constant slope, which equals the marginal revenue $\bar{p}$, and the R&D investment curve has a decreasing slope, which equals the marginal R&D investment *i*_*q*_. When the R&D investment is 0, the demand is *q*_0_, and the total revenue is *π*_0_.

From Fig 2A, we can get that when $i_{q}|{}_{i=0}>\bar{p}$, there is a unique R&D investment that makes the firm obtain minimum profits, and the unique non-zero R&D investment *i*_1_ that makes the firm’s profits equal to *π*_0_. If *i*_1_ ≤ *i*^max^, the firm’s optimal R&D investment strategy is i* = *i*^max^, otherwise i* = 0. From Fig 2B, we can get that when $i_{q}|{}_{i=0}\leq \bar{p}$ the slope of the R&D investment curve is always lower than marginal revenue and the firm’s optimal R&D investment strategy is i* = *i*^max^. In sum, the optimal R&D investment strategies with *i*_*qq*_ < 0 are

$$i^{*}=\{\begin{array}{ccccc} i^{\text{max}} & i_{q}|{}_{i=0}>\bar{p} & and & i_{1}\leq i^{\text{max}} & or\;i_{q}|{}_{i=0}\leq \bar{p}\\ 0 & i_{q}|{}_{i=0}>\bar{p} & and & i_{1}>i^{\text{max}} & \end{array}$$ (4)

Q.E.D.

On the one hand, when the marginal R&D investment is always lower than marginal revenue, the firm’s wise choice is to invest its entire R&D funds to get as much profits as possible. On the other hand, the firm’s marginal R&D investment is no lower than the marginal revenue at first and then smaller than it. With increasing R&D investment, the firm will lose money first and then make money. Therefore, if the R&D funds are sufficient to make the firm benefit, it invests all for as much profits as possible; otherwise, it does not conduct R&D to avoid losses.

Furthermore, it is easy to find that when the firm’s marginal R&D investment decreases, as long as there is enough R&D investment, it can benefit from R&D. In other words, the financial constraints hinder NEV firms from increasing R&D investment in this situation.

We then solve the NEV firm’s optimal R&D investment strategies when its marginal R&D investment is increasing, or *i*_*qq*_ > 0. Let *i*_2_ be the optimal R&D investment, and we can get proposition 2 as follows.

**Proposition 2.** In the case of *i*_*qq*_ > 0:

(a) When the initial value of marginal R&D investment is lower than the marginal revenue, if the firm’s R&D funds are sufficient, the optimal R&D investment strategy is making the marginal R&D investment equal to the marginal revenue, otherwise the entire R&D funds;
(b) When the initial value of marginal R&D investment is no lower than the marginal revenue, the optimal R&D investment strategy is zero.

**Fig 3:**
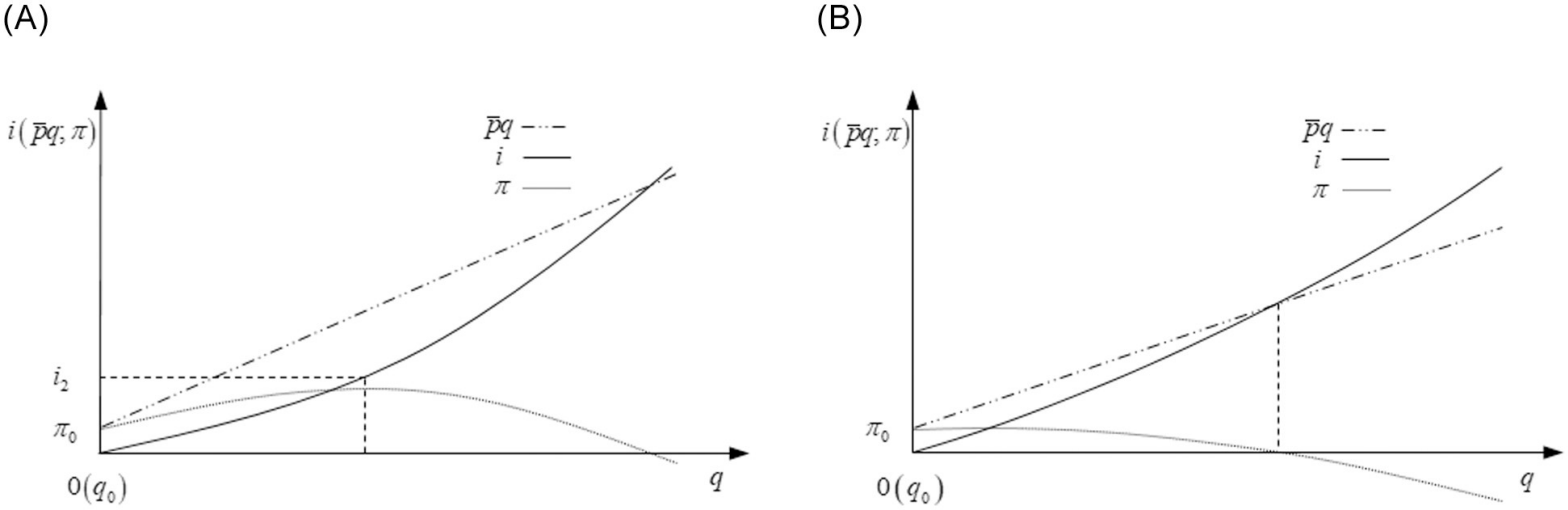
A. Changes in profits and revenue when *i*_*qq*_ > 0 and $i_{q}|{}_{i=0}<\bar{p}$. B. Changes in profits and revenue when *i*_*qq*_ > 0 and $i_{q}|{}_{i=0}\geq \bar{p}$.

**Proof of Proposition 2.** Unlike proposition 1, the R&D investment curve has an increasing slope in Fig 3A and 3B, that is, *i*_*qq*_ > 0.

From Fig 3A, if $i_{q}|{}_{i=0}<\bar{p}$, the firm obtains its unique maximum profits when $i_{q}|{}_{i=i_{1}}=\bar{p}$, and the optimal R&D investment strategy is i* = *i*^max^ if *i*_1_ ≥ *i*^max^, otherwise, its optimal R&D investment strategy is i* = *i*_1_.

From Fig 3B, if $i_{q}|{}_{i=0}\geq \bar{p}$, the slope of the R&D investment curve is always higher than marginal revenue, and the firm’s unique maximum profits at i* = 0.

In sum, the optimal R&D investment strategies with *i*_*qq*_ > 0 are

$$i^{*}=\{\begin{array}{cccc} i^{\text{max}} & i_{q}|{}_{i=0}<\bar{p} & and & i_{1}\geq i^{\text{max}}\\ i_{1} & i_{q}|{}_{i=0}<\bar{p} & and & i_{1}<i^{\text{max}}\\ 0 & i_{q}|{}_{i=0}\geq \bar{p} & & \end{array}$$ (5)

Q.E.D.

On the one hand, when the firm’s marginal R&D investment is lower than the marginal revenue first and then higher, the firm can obtain maximum profits by making its marginal R&D investment equal to marginal revenue. However, the firm’s R&D funds are limited. If the firm can build its marginal R&D investment equal to marginal revenue, it will invest the required R&D funding; otherwise, it will spend its entire R&D funding to get as much profits as possible. On the other hand, when the firm’s marginal R&D investment is no lower than the marginal revenue, the marginal R&D investment is always higher than marginal revenue, and the wise choice is not R&D.

Furthermore, we can find that when the firm’s marginal R&D investment increases, the lack of positive marginal profits prevents the firm from increasing R&D investment firstly. That is, the firm cannot always obtain positive marginal profits, or can not achieve positive marginal profits when the marginal R&D investment is higher. Then, even if the profit incentive is sufficient, financial constraints may become the second obstacle.

### Crowding-in effect

In this section, we solve the crowding-in effect of product subsidy by comparing the NEV firm’s optimal R&D investment strategies with and without the subsidy. The superscript “^*N*^” of variables implies that the government doesn’t provide product subsidies.

First, referring to the previous section, we solve the firm’s optimal R&D investment strategies without product subsidy. It is easy to explain when there is *i*_*qq*_ < 0, the optimal R&D investment strategies are

$$i^{N*}=\{\begin{array}{cccc} \text{0} & i_{q}|{}_{i=0}>p & and & i_{2}^{N}>i^{\text{max}}\\ i^{\text{max}} & i_{q}|{}_{i=0}>p & and & i_{2}^{N}\leq i^{\text{max}}\;or\;i_{q}|{}_{i=0}\leq p \end{array}$$ (6)

when there is *i*_*qq*_ > 0, the optimal R&D investment strategies are

$$i^{N*}=\{\begin{array}{cccc} i^{\text{max}} & i_{q}|{}_{i=0}<p & and & i_{1}^{N}\geq i^{\text{max}}\\ i_{1}^{N} & i_{q}|{}_{i=0}<p & and & i_{1}^{N}<i^{\text{max}}\\ 0 & i_{q}|{}_{i=0}\geq p & & \end{array}$$ (7)

Before comparing the optimal R&D investment strategies, two pairs of values for the optimal R&D investment, that is $i_{1}^{N}$ and *i*_1_, and $i_{2}^{N}$ and *i*_2_ need to be compared.

**Lemma 2.** (a) When *i*_*qq*_ < 0, there is $i_{1}<i_{1}^{N}$; (b) When *i*_*qq*_ > 0, there is $i_{2}>i_{2}^{N}$;.

**Proof of Lemma 2.** When *i*_*qq*_ < 0, we know that $i_{1}^{N}$ satisfies $\pi^{N}|{}_{i^{N*}=i_{1}^{N}}=pq\left(i_{1}^{N}\right)-i_{1}^{N}=pq_{0}$ and *i*_1_ satisfies $\pi |{}_{i^{*}=i_{1}}=\bar{p}q\left(i_{1}\right)-i_{1}=\bar{p}q_{0}$.

Then, *pq*(*i*_1_) − *i*_1_ = *π*_0_ = *pq*_0_ − *s*(*q*(*i*_1_) − *q*_0_). As *q*(*i*_1_) − *q*_0_ > 0, $pq\left(i_{1}\right)-i_{1}<pq\left(i_{1}^{N}\right)-i_{1}^{N}$.

Further, from *π*^*N*^ = *pq*(*i*^*N*^) − *i*^*N*^, *i*_*q*_ > 0, and *i*_*qq*_ < 0, if *i*_*q*_ < *p*, the bigger *π*^*N*^, the bigger the corresponding *i*^*N*^. As $i_{q}|{}_{i^{*}=i_{1}}>\bar{p}$ and $i_{q}^{N}|{}_{i^{N*}=i_{1}^{N}}>p$, we can get $i_{1}<i_{1}^{N}$.

When *i*_*qq*_ > 0, we know that $i_{2}^{N}$ satisfies $i_{q}|{}_{i=i_{2}^{N}}=p$ and *i*_2_ satisfies $i_{q}|{}_{i=i_{2}^{N}}=\bar{p}$. As $i_{q}|{}_{i=i_{2}^{N}}=p<\bar{p}=i_{q}|{}_{i=i_{2}^{N}}$ and *i*_*qq*_ > 0, $i_{2}^{N}<i_{2}$.

Q.E.D.

On the one hand, when the firm’s marginal R&D investment is decreasing, and its initial value of marginal R&D investment is higher than the marginal revenue, the firm can achieve the profits that the firm conducts no R&D with an R&D investment amount. As product subsidies increase each NEV’s marginal gains, the firm can benefit from R&D with a lower R&D investment amount than without product subsidy.

On the other hand, when the firm’s marginal R&D investment is increasing, and its initial value of marginal R&D investment is lower than the marginal revenue, the firm has the optimal R&D investment to achieve its maximum profits. As product subsidies increase the firm’s marginal revenue, it can gain positive profits at a higher marginal R&D investment. In other words, the firm gets higher maximum profits at a higher optimal R&D investment amount.

We then solve the impact of product subsidy on the firm’s R&D investment when *i*_*qq*_ < 0 and *i*_*qq*_ > 0. Let Δ be the difference between the optimal R&D investment with and without product subsidy, and we can get propositions 3 and 4 as follows.

**Proposition 3.** In the case of *i*_*qq*_ < 0:

(a) Only if the firm cannot benefit from R&D due to financial constraints, and the unit subsidy is large enough, it has a crowding-in effect;
(b) If there is a crowding-in effect, it always equals the firm’s entire R&D funds.

**Proof of Proposition 3.** When *i*_*qq*_ < 0, (4) and (6) are compared. The results are shown in Table 1.

Q.E.D.

**Table 1:** Crowding-in effect when *i*_*qq*_ < 0.

R&D investment without product subsidy	R&D investment with product subsidy	The crowding-in effect
$p<i_{q}|{}_{i=0}\;and\;i^{\text{max}}<i_{1}^{N}$	$\bar{p}\leq i_{q}|{}_{i=0}\;and\;i^{\text{max}}<i_{1}<i_{1}^{N}$	Δ = 0
$p<i_{q}|{}_{i=0}\;and\;i^{\text{max}}<i_{1}^{N}$	$\bar{p}\leq i_{q}|{}_{i=0}\;and\;i_{1}\leq i^{\text{max}}<i_{1}^{N}$	Δ = *i*^max^
$p<i_{q}|{}_{i=0}\;and\;i^{\text{max}}<i_{1}^{N}$	$\bar{p}>i_{q}|{}_{i=0}$	Δ = *i*^max^
$p<i_{q}|{}_{i=0}\;and\;i_{1}^{N}\leq i^{\text{max}}$		Δ = 0
*p* ≥ *i*_*q*_|_*i*=0_		Δ = 0

Proposition 3 shows that, as we discovered from proposition 1, the NEV firm does not increase R&D investment due to financial constraints in this situation. Only when the firm does not conduct R&D, and the unit subsidy is large enough to reduce the profitable R&D investment threshold, which indirectly affects the firm’s financial constraints, the firm switches to conduct R&D. To get the most profits, the firm will always invest its entire R&D funds, which is the second and third cases in Table 1. In the first case, the unit subsidy is not significant enough to have a crowding-in effect.

On the other hand, if the firm has invested all, the product subsidy is ineffective, which is the fourth and fifth cases in Table 1.

Furthermore, for product subsidies to work, it is reasonable to only provide product subsidies to NEV firms that did not conduct R&D when its marginal R&D investment is decreasing. Of course, it must also be noted that the unit subsidy must be large enough. On the contrary, if the firm has carried out R&D before the subsidy, it is reasonable to help it solve the R&D financial constraints.

**Proposition 4.** In the case of *i*_*qq*_ > 0:

(a) If the firm cannot benefit from R&D without product subsidies, only if the unit subsidy is large enough is a crowding-in effect;
(b) If the firm has conducted R&D without product subsidies, there is always a crowding-in effect except it has no remaining R&D funds;
(c) If there is a crowding-in effect, it increases with the unit subsidy until the firm invests its entire R&D funds.

**Proof of Proposition 4.** When *i*_*qq*_ > 0, (5) and (7) are compared. The results are shown in Table 2.

Q.E.D.

**Table 2:** Crowding-in effect when *i*_*qq*_ > 0.

R&D investment without product subsidy	R&D investment with product subsidy	The crowding-in effect
*p* ≤ *i*_*q*_|_*i*=0_	$\bar{p}\leq i_{q}|{}_{i=0}$	Δ = 0
*p* ≤ *i*_*q*_|_*i*=0_	$\bar{p}>i_{q}|{}_{i=0}\;and\;i_{2}<i^{\text{max}}$	Δ = *i*_2_
*p* ≤ *i*_*q*_|_*i*=0_	$\bar{p}>i_{q}|{}_{i=0}\;and\;i_{2}\geq i^{\text{max}}$	Δ = *i*^max^
$p>i_{q}|{}_{i=0}\;and\;i_{2}^{N}<i^{\text{max}}$	$\bar{p}>i_{q}|{}_{i=0}\;and\;i_{2}^{N}<i_{2}<i^{\text{max}}$	$\Delta =i_{2}-i_{2}^{N}$
$p>i_{q}|{}_{i=0}\;and\;i_{2}^{N}<i^{\text{max}}$	$\bar{p}>i_{q}|{}_{i=0}\;and\;i_{2}^{N}<i^{\text{max}}\leq i_{2}$	$\Delta =i^{\text{max}}-i_{2}^{N}$
$p>i_{q}|{}_{i=0}\;and\;i_{2}^{N}\geq i^{\text{max}}$	$\bar{p}>i_{q}|{}_{i=0}\;and\;i^{\text{max}}<i_{2}^{N}<i_{2}$	Δ = 0

Proposition 4 shows that when the NEV firm’s marginal R&D investment increases, both the lack of positive marginal profits and R&D financial constraints may prevent the firm from increasing R&D investment. In case 2–5 of Table 2, product subsidy enables the firm to achieve positive marginal profits, or to obtain positive marginal gains at a higher marginal R&D investment. Then, the firm increases its R&D investment with product subsidies. Regardless of financial constraints, the higher the unit subsidy, the greater a crowding-in effect. However, the crowding-in effect or its increment are all limited by the firm’s financial constraints, and will not exceed the firm’s entire R&D funds, such as the third case and the fifth case in Table 2. The unit subsidy is are not significant enough to incentivize companies to increase R&D investment in the first case of Table 2. In the last case, the firm has invested all, so that product subsidy can not have a crowding-in effect.

Furthermore, if the NEV firm has an increasing marginal R&D investment, product subsidy generally has a crowding-in effect. However, there are still some problems that should be noticed. On the one hand, if the firm is not willing to carry out R&D before product subsidy, only if significant enough unit subsidies are effective. On the other hand, before the government provides product subsidy, it is necessary to understand whether the NEV firm still has unexpended R&D funds. In other words, only when profit incentives match funds supports can product subsidy achieve the desired effect.

### Numerical analyses

In this section, we use choose an arbitrary quantity test our model. Let *a*(*i*) = *e*^*vi*^, where *v* is the firm’s R&D capability coefficient and *a*_0_ = *a*(0) = 1. Readily shown, *i*_*q*_ > 0 and *i*_*qq*_ < 0, which indicates that the firm has a decreasing marginal R&D investment. Moreover, the marginal R&D investment function is $i_{q}=a_{i}^{-1}=v^{-1}e^{-vi}$, where *i*_*q*_|_*i*=0_ = *v*^−1^. The firm’s profits function is *π*(*i*) = (*p* + *s*)*e*^*vi*^ − *i*.

Where *i*^max^ = 1.9, *x* = 0.5 and *p* = 0.5, we can calculate the initial value of marginal R&D investment as *i*_*q*_|_*i*=0_ = 2. From proposition 1, we know that $i^{N*}=0$. Thereafter, we show that the changes of the crowding-in effect of the unit subsidy increases from 0 to 1.

**Fig 4:**
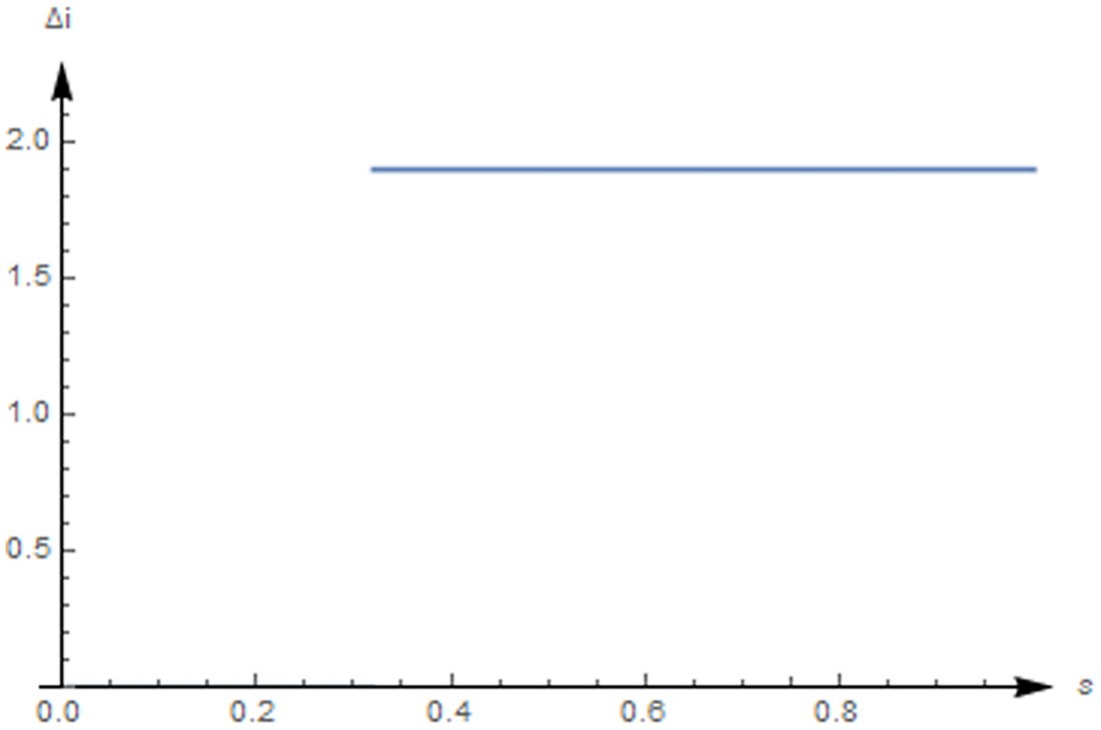
The crowding-in effect when *i*_*qq*_ < 0.

In Fig 4, one case shows that the NEV firm has a decreasing marginal R&D investment, and its initial value of marginal R&D investment is higher than the marginal revenue. More specifically, establish a unit subsidy threshold, whereby *s* = 0.32, which, in turn, determines whether the crowding-in effect is present. Firstly, in this case a unit subsidy no lower than 0.32, shows that the crowding-in effect is present. Secondly, a unit subsidy no lower than 0.32 indicates that the firm ordinarily invests its R&D funds in their entirety.

We then take into consideration that the NEV firm has an increasing marginal R&D investment. Let $a\left(i\right)=\bar{x}-e^{-vi}$, where $\bar{x}$ is the quality of ICEVs and $a_{0}=\bar{x}-1$. Readily shown, *i*_*q*_ > 0 and *i*_*qq*_ > 0. Moreover, the marginal R&D investment function is $i_{q}=a_{i}^{-1}=v^{-1}e^{vi}$, where *i*_*q*_|_*i*=0_ = *v*^−1^. The firm’s profits function is $\pi \left(i\right)=\left(p+s\right)\left(\bar{x}-e^{-vi}\right)-i$.

Let *i*^max^ = 1.9, *v* = 0.5, and *p* = 1 or *p* = 3. Easy to get *i*_*q*_|_*i*=0_ = 2. From proposition 2, we know that $i^{N*}=0$ or $i^{N*}=0.81$. Then, we show the changes in the crowding-in effect when the unit subsidy increases from 0 to 5.

**Fig 5:**
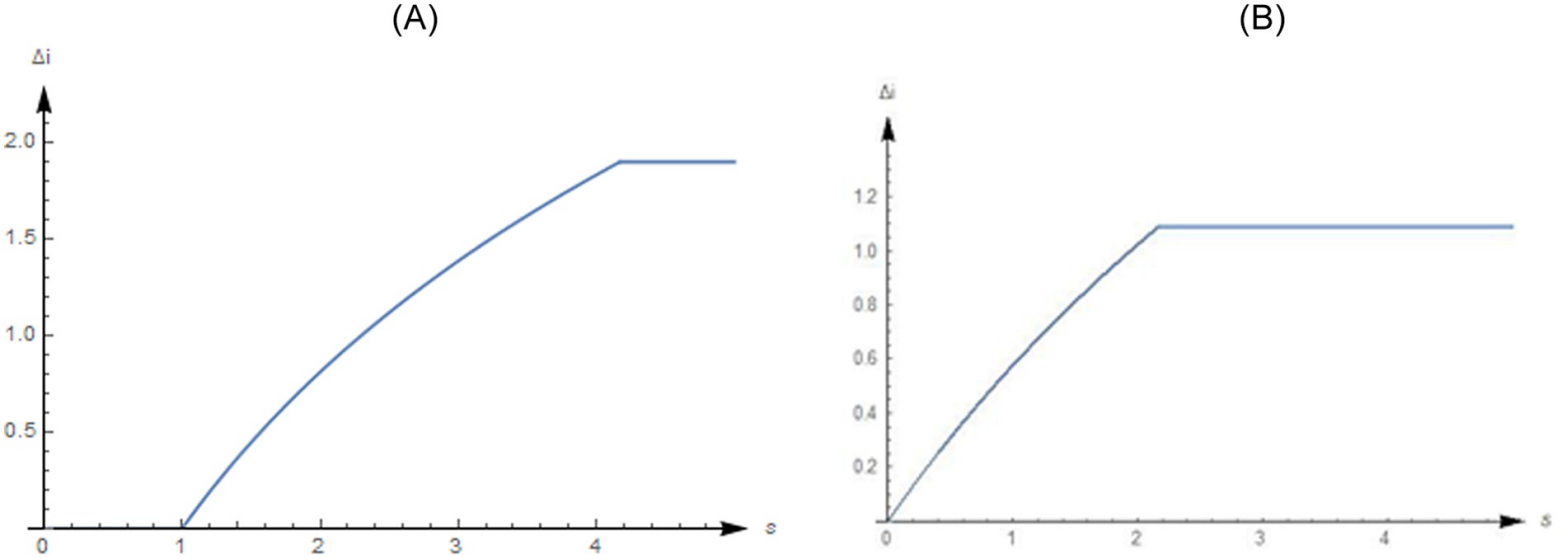
A. Crowding-in effect when *i*_*qq*_ > 0 and *p* = 1. B. Crowding-in effect when *i*_*qq*_ > 0 and *p* = 3.

In Fig 5A, the NEV firm has an increasing marginal R&D investment. The initial value of marginal R&D investment of the firm is higher than the marginal revenue, that is, *i*_*q*_|_*i*=0_ = 2 > 1 = *p*. We can see that the crowding-in effect doesn’t exist when the unit subsidy is lower than 1. After the unit subsidy exceeds 1, the crowding-in effect increases with it from 1 to 4.17 first, and then equals *i*^max^ = 1.9. This means that only if the unit subsidy is large enough, which should be higher than 1 in this case, the product subsidy has a crowding-in effect. However, if the unit subsidy is higher than 4.17, the crowding-in effect will remain unchanged. The reason for this is that the firm will ordinarily invest its entire R&D budget subject to financial constraints.

In Fig 5B, shows that the NEV firm can benefit from R&D without a product subsidy, and the crowding-in effect will increase as the unit subsidy increases from 0 to 2.17 until the firm invests its whole available R&D budget.

## Conclusions

Given that the early adopter group’s consumers have an increasing density on acceptable quality thresholds, we proposed a vertical quality differentiation model of product R&D with subsidies. The impact of product subsidies on the NEV firm’s R&D investment is discussed. We hope to have shed some light on how to rationally deploy and improve government product subsidy policies.

The NEV firms’ marginal R&D investment supported by increases in sales may fluctuate according to the increasing density in early adopters with minimum acceptable quality thresholds. We show that firms with a decreasing marginal R&D investment are either reluctant to undertake R&D, or unable to increase their investment in R&D due to budget constraints. At best, firms that are unable to conduct their own R&D can benefit from the granting of significant unit product subsidies, whereby these produce a crowding-in affect equivalent to their entire R&D budgets. By contrast, firms that are constrained by marginal increases in R&D investment, inevitably face the dilemma of lower profits against increases in R&D investments. In general, the crowding-in effect from product subsidies result in higher company profit margins. More specifically, firms who rely on subsidies to conduct R&D will only do so with adequate unit grants. Thus, increases in unit subsidies and the crowding-in effect are interdependent. However, the aforementioned conclusions are limited to those firms that are financially constrained by having spent their entire R&D budget, and are unable to make use of subsidies to produce or increase a crowding-in effect.

Furthermore, governments are advised to consider the quality preferences of early adopter groups. Based on a situational analysis, subsidies should be granted to NEV firms, provided there is a both a decrease in their marginal R&D investment, and they rely on subsidies to conduct R&D. The appropriate amount of the unit subsidies should be calculated accordingly at the equivalent baseline formation of a crowding-in effect. The reasonable alternative hereto is to replace the product subsidy policy with a funding support policy. In contrast, if firms increase their marginal R&D investment, it becomes necessary to achieve or improve the crowding-in effect by adjusting the unit subsidies. However, if firms are financially constrained by having spent their entire R&D budgets, the formation of a greater crowding-in effect requires facilitation through a funding support policy.

## Supporting information

S1 File (DOCX)
