# Supplementary material for: Impact of product subsidies on R&D investment for new energy vehicle firms: Considering quality preference of the early adopter group
Source: PLoS One. 2020 Jul 31;15(7):e0236626. doi: 10.1371/journal.pone.0236626 (PMC7394409; doi:10.1371/journal.pone.0236626)
Supplement: S1 File — (DOCX) [file pone.0236626.s001.docx]

**[Title Page]**

**Impact of product subsidies on R&D investment for new energy vehicle firms: Considering quality preference of the early adopter group**

Short title: Impact of product subsidies on new energy vehicle R&D

**Weidong Meng**^1^**, Ye Wang**^1^**, Yuyu Li**^2^ **and Bo Huang**^1*^

^1^ **School of Economics and Business Administration, Chongqing University, Chongqing, China**

^2^ **School of Economics and Management, Chongqing Normal University, Chongqing, China**

*** Correspondence author**

**E-mail:** [**huangbo@cqu.edu.cn**](mailto:huangbo@cqu.edu.cn) **(BH)**

**Abstract**

Different consumer groups accept new energy vehicles sequentially from the perspective of innovation diffusion theory, and the early adopter group has recently been identified. By assuming that that the density of early adopters is increasing at minimum acceptable quality thresholds, this paper proposes a vertical quality differentiation model of product R&D with product subsidies. The impact of product subsidies on the R&D investment of new energy vehicle firms is discussed. We show that the early adopters’ characteristics may affect the stagnant marginal R&D investment of new energy vehicle firms by increasing sales, which determines the impact mechanism of product subsidies. For firms with decreasing marginal R&D investments, insufficient R&D investments result from financial constraints. If insufficient R&D resources deter firms from conducting R&D, substantial unit subsidies invariably incentivize firms to spend their entire R&D budget. Firms with increasing marginal R&D investments, insufficient R&D profits, or financial constraints are prevented from increasing R&D investment. Product subsidies generally have a crowding-in effect on firms not subject to financial constraints, and this effect increases with the unit subsidy. However, the existence of a crowding-in effect may require sufficiently large unit subsidies. In both situations, product subsidies cannot modulate financial constraints if the firm has spent its entire R&D budget. In the first situation, we also show that product subsidies should be replaced by a funding support policy. In contrast, the second situation shows that a funding support policy should be coordinated with product subsidies.

**Introduction**

Recently, new energy vehicle (NEV) development has captured the interest of both the public and private sectors, which is of great significance for the sustainable development of energy and the environment [1-3]. Though it seems unrealistic for NEVs to replace internal combustion engine vehicles (ICEVs) now because their technologies (quality) still need considerable improvement [4, 5]. To encourage strengthening R&D, some governments have provided strong R&D subsidy policies for NEV firms [6-9]. China, for example, has spent more than 1000 billion Chinese yuan up to now on a product subsidy policy for firms from the central government [11-14]. The policy did not achieve the government’s expected targets [9, 15]. Therefore, this paper aims to provide some useful suggestions for this timely and critical issue, on how to encourage NEV firms to increase their R&D investment by product subsidies for firms.

The government’s considerations in funding incentives for private R&D is usually guided by ancillary benefits, leading to underfunded R&D investment in private firms [16, 17]. However, the conclusions reached by a large number of similar studies do not indicate the expected effects of subsidies the R&D investment of firms [18-20]. These inconsistencies are easily identifiable, among others a crowding-in effect, a crowding-out effect, no crowding-out effect, and mixed effect [21-24]. A fundamental reason for inconsistencies in the policies guiding the granting of subsidies is that they inevitably are not only specific to the economic environment, but also applicable to distinct channels, and influenced by numerous factors [24, 25].

Seminal in this field is to investigate which aspects are effective in the granting of subsidies. Hassine and Mathieu [26] found that government subsidies create a significant leverage eﬀect, and firms inside the industry clusters receive more R&D subsidies and invest more R&D funds comparing with firms outside clusters. Bai et al. [27] found that R&D subsidies increase the green innovation of energy-intensive firms, where the impact is more robust for state-owned firms and SMEs. Yu et al. [28] found that the relationship between the effect of government subsidies and renewable energy firms’ R&D intention presents an inverted-U shape, which is further moderated by the attributes of firm ownership. Yang and Xiao [29] considered that the government provides subsidies for manufacturers with a green level floor and, no matter how high this floor is, retailers as leaders in the supply chain will create higher R&D investment than manufacturers as leaders. Howell [30] found that an early-stage R&D subsidy positive impacts on the firm’s innovation, though these effects are more forceful for firms with financial constraints.

In this field of research, there is a lack of those that study NEV firms by focusing on how they are uniquely affected by subsidies. The dearth of studies we examine now may be attributed to insufficient data, or the classification of NEV firms as automotive, manufacturing, or environmental R&D [31-33]. Jiang et al. [31] found that government subsidies will increase the NEV firms’ R&D investment, which is significant for assembly firms but not for supporting firms. Xiong, Fan, and Liu [34] found that appropriate fiscal subsidies have an incentive effect for manufacturers’ R&D investment, and too many subsidies will have a negative impact. Strategic decision-making elements such as the executive shareholding ratio, ownership concentration, and proportion of independent directors have a significant impact on the incentive effect of fiscal subsidy in R&D investment. Liu and Zhao [35] found that a government’s subsidy policy can effectively promote the technological progress of NEV firms. However, market end subsidies shall be dropped out timely while the technology end subsidies should be strengthened.

the two aspects should be of concern in the NEV market, namely the phenomenon of consumer grouping, and an existing acceptable quality threshold for early adopters. These aspects also show characteristic features. While NEVs are new, environmentally friendly products, vehicles in themselves are a longstanding means of human transportation. Rogers [36] posits that the five categories of early adopters of green NEVs follow a temporal sequence in adopting innovation, as illustrated in Fig 1. Furthermore, many scholars recognized that the current consumer group is the early adopters in related research [37-40].

**Fig 1. Categorizing adopters according to a new product life cycle.**

Furthermore, these aforementioned researchers also established that the main concerns of early adopters are price, environmental friendliness and energy efficiency, and the quality of NEVs [37-40]. The quality primarily refers to NEV performance specifications, such as battery range and charging. A number of researchers affirm that discounts, or environmental friendliness and energy efficiency are the most prominent factors contributing to the growth of the NEVs market [39, 40]. However, these findings are negated by the development of China’s NEV market. Despite subsidized NEV products, their price being competitively with ICEVs, their market share currently accounts for less than 3% of the entire vehicle market [41]. In contrast, the concerns of consumer about NEV quality, such as “range anxiety,” “charging anxiety,” imply that the minimum acceptable for quality threshold for early adopters is subjective, especially when comparing ICEV performances.

Ongoing studies into the purchase decision-making process of NEV consumers supports this view. [42-47]. Hackbarth and Madlener [48] found that the quality of alternative fuel vehicles has to meet some minimum requirements for consumers who are willing to pay considerable amounts for the improvement of alternative fuel vehicles on driving range, fuel availability, and recharging time. Neaimeh et al. [49] found that if the distance of a journey is above the single-charge range of the battery electric vehicle, taking several hours to charge with standard chargers is unacceptable for consumers. Noel et al. [50] found that although electric vehicles have greatly improved in recent years, common barriers like range and charging infrastructure continue to persist.

What distinguishes this study is that we consider group of early adopters who have a high minimum threshold for acceptable quality. In general, R&D has had a peripheral effect on the improvement of quality, whereas NEV firms have marginally increased their R&D investment by increasing sales. Due consideration of the characteristics of early adopters could ostensibly lead to arresting the growing trend of marginal R&D investment, and conceivably a reversal. In response, we analyzed the product subsidy mechanism and its impact on the NEV firm’s R&D investment by comparing the fluctuations in its marginal R&D investment. Our findings indicate that product subsidy has a crowding-in effect on NEV firm that faces insufficient profits but have limited impact on inadequate R&D investment due to funds constraints. In particular, where the NEV firm has a decreasing marginal R&D investment and does not conduct R&D as limited R&D funds, product subsidy can reduce the profitable R&D investment threshold. That is, the product subsidy may indirectly impact the NEV firm’s financial constraints and result in a crowding effect on its R&D investment. Based on these findings, governments need to adopt a rational policy in deploying and optimizing product subsidies.

The remainder of the paper is organized as follows. In section 2, we describe the vertical differentiation model of product R&D for an NEV firm with product subsidies. The optimal R&D investment strategies and the crowding-in effect of product subsidies are obtained in sections 3 and 4. The numerical analysis is carried out in section 5, and the conclusions are given in section 6.

**The model**

This study investigates one type of NEV and ICEV on the market, provided by one NEV firm and several ICEV firms, respectively. Whereas all consumers are regarded as potential buyers of ICEVs, a selected few are regarded as “the early adopters” of NEVs [36]. Our discussion below focuses on those early adopters.

The early adopters display acceptable quality threshold range $\left[\underline{x},\bar{x}\right]$, uniformly distributed, and where $\bar{x}$ is the quality of ICEVs. They distribute across this interval with increasing density $g\left(x\right)dx$. We assume, that if the energy-efficiency and environmental friendliness of the NEV exceeds their acceptable quality threshold, the consumer will choose to buy an NEV rather than an ICEV. Similarly, where ICEVs are of higher quality than NEVs, buyers are reluctant to pay more for an NEV. Moreover, when it is assumed that the ICEV market is competitively at priced at$p$, NEV prices, irrespectively, should not exceed this.

Further, consider how NEV consumers make purchase decisions. Referring to a version of the standard quality differentiation model [51-53], assume a consumer either buys an NEV or does not buy any. Then, under the identical budget constraint of NEV consumers $p^{NEV}+r\leq y$, give the early adopter’s utility function is:

$U\left(x,r;x^{n}\right)=r+\theta x\text{ }if\text{ }x\geq x^{n}\text{ }or\text{ }U\left(x,r;x^{n}\right)=r\text{ }if\text{ }x<x^{n}$,

where $x_{n}$ is the acceptable quality threshold of consumer $n$, $p^{NEV}$ is the NEV price, $r$ is a numeraire good, $y$ is the consumer income, $\theta$ is the price coefficient of unit quality, and $\theta\bar{x}\leq p$. From (1) and its derivative $U_{x}=\theta$ (the subscripts here and below denote a derivation except those in numerical form), it is clear that a consumer with utility maximization is always willing to pay for additional quality improvement if only the NEV reaches the threshold. Moreover, with a quality $x$ and a price below $p$, the potential consumer number who will buy an NEV is $a\left(x\right)=\int_{\underline{x}}^{x} g\left(x\right)dx$, which satisfies $a_{x}>0$ and $a_{xx}>0$. This implies that the closer an NEV is to ICEV quality, in driving range and recharge time for example, the number of potential consumers who can accept them increases faster. It is worth noting that we use a continuous function $a\left(x\right)$ instead of a discrete one for simplicity, but this does not affect our analysis and results.

In addition to the characteristics of consumers, the characteristics of the NEV firm should also be recognized. The firm has limited R&D funding $i^{\max}$, which is far from enough to bring NEV quality to $\bar{x}$. It also has a technology reserve to improve the NEV quality to $x_{0}$, which satisfies $x_{0}>\underline{x}$. Moreover, with different levels of R&D investment $i$, the firm can make NEVs reach different levels of quality $x$, which satisfies $x=x\left(i\right)$, $x\geq x\left(0\right)=x_{0}$, $x_{i}>0$ and $x_{ii}<0$.

Then, consider a three-stage decision-making process. In the first stage, the government sets the standard of product subsidy, which is the subsidy amount the firm will receive for each NEV sold. In the second stage, the firm makes R&D or R&D investment decisions. In the third stage, the firm makes NEV pricing decisions when the ICEV is priced at $p$. In fact, the NEV pricing issue does not require complicated analysis later, as the firm will always price NEVs at $p$. The reason is simple; if only the NEV quality reaches a consumer’s threshold, he or she has a reserved price $p$.Therefore, only if the firm prices NEVs at $p$, it can maximize its profits. Moreover, since the fixed marginal production cost will not affect our result, it will be simplified to zero below.

Accordingly, we get the firm’s demand function as:

$q\left(i\right)=a\left[x\left(i\right)\right]=\int_{\underline{x}}^{x} g\left(x\right)dx$,

where $q_{0}=q\left(0\right)=a\left(x_{0}\right)\left|_{i=0}\right.=\int_{\underline{x}}^{x_{0}} g\left(x\right)dx>0$, and the profit function as:

$\pi\left(i\right)=\left(p+s\right)q\left(i\right)-i$,

where $\pi_{0}=\pi\left(0\right)=q_{0}\left(p+s\right)$, $\left(p+s\right)q\left(i\right)$ and $\bar{p}=p+s$ are the firm’s revenue and revenue per NEV.

**Optimal R&D investment strategies**

In this section, we will solve the optimal R&D investment strategies of the NEV firm with product subsidy. It is worth noting that a graphical method is adopted for solving the strategies, which allows us to explain our findings more realistically with the concept of marginal R&D investment. Before solving, we will analyze the change in the firm’s marginal R&D investment with the growth of its sales.

**Lemma 1.** When $x_{i}>0$, $x_{ii}<0$, $a_{x}>0$ and $a_{xx}>0$, the NEV firm’s marginal R&D investment with sales can be decreasing $i_{qq}<0$ or increasing $i_{qq}>0$.

**Proof of Lemma 1.** As $a_{x}>0$ and $x_{i}>0$, we can get $a_{i}=a_{x}x_{i}>0$. And as $a_{xx}>0$ and $x_{ii}<0$, we can get that $a_{ii}=a_{xx}x_{i}^{2}+a_{x}x_{ii}$ can be positive or negative. From (2), we know that $q_{ii}=a_{ii}$ can be negative or positive.

Therefore, according to the derivative rule of inverse functions, we know that $i_{q}=\left(q_{i}\right)^{-1}>0$ and $i_{qq}$ has the opposite sign with $q_{ii}$.

Q.E.D.

In general, since the R&D of the NEV firm has a marginal diminishing effect on quality improvement, it will have increasing marginal R&D investment. However, when considering the early adopter group’s consumers have an increasing density on acceptable quality thresholds, increasing pressure on the firm’s marginal R&D investment will be eased. If the early adopters’ distribution on acceptable quality thresholds has a significant enough impact, the firm has a decreasing marginal R&D investment instead; otherwise, it still has an increasing marginal R&D investment.

First, we solve the NEV firm’s optimal R&D investment strategies when its marginal R&D investment is decreasing, or $i_{qq}<0$. Let $i_{1}$ be the R&D investment, which makes the firm’s profits equal to $\pi_{0}$, and we can get proposition 1 as follows.

**Proposition 1**. In the case of $i_{qq}<0$:

(a) When the initial value of marginal R&D investment is no higher than the marginal revenue, and when the initial value of marginal R&D investment is higher than the marginal revenue and the firm has enough R&D funds, the optimal R&D investment strategy is the entire R&D funds;

(b) Otherwise, the optimal R&D investment strategy is zero.

**Proof of Proposition 1**. From Figs 2A and 2B, we know the revenue curve has a constant slope, which equals to the marginal revenue $\bar{p}$, and the R&D investment curve has a decreasing slope, which equals to the marginal R&D investment $i_{q}$. When the R&D investment is 0, the demand is $q_{0}$ and the total revenue is $\pi_{0}$.

From Fig 2A, we can get that when $i_{q}\left|_{i=0}\right.>\bar{p}$ there is a unique R&D investment that makes the firm obtain minimum profits, and the unique non-zero R&D investment $i_{1}$ that makes the firm’s profits equal to $\pi_{0}$. If $i_{1}\leq i^{\max}$, the firm’s optimal R&D investment strategy is $i^{*}=i^{\max}$, otherwise $i^{*}=0$.

**Fig 2A. Changes in profits and revenue when $i_{qq}<0$ and** $i_{q}\left|_{i=0}\right.>\bar{p}$**.**

From Fig 2B, we can get that when $i_{q}\left|_{i=0}\right.\leq\bar{p}$ the slope of the R&D investment curve is always lower than marginal revenue and the firm’s optimal R&D investment strategy is $i^{*}=i^{\max}$.

**Fig 2B. Changes in profits and revenue when $i_{qq}<0$ and** $i_{q}\left|_{i=0}\right.\leq\bar{p}$**.**

In sum, the optimal R&D investment strategies with $i_{qq}<0$ are

$i^{*}=\left\{\begin{matrix} i^{\max}\text{ }i_{q}\left|_{i=0}\right.>\bar{p}\text{ }and\text{ }i_{1}\leq i^{\max}\text{ }or\text{ }i_{q}\left|_{i=0}\right.\leq\bar{p}\text{ } \\ \text{0 }i_{q}\left|_{i=0}\right.>\bar{p}\text{ }and\text{ }i_{1}>i^{\max}\text{ } \end{matrix}\right.$.

Q.E.D.

Proposition 1 shows on one hand, when the marginal R&D investment is always lower than marginal revenue, the firm’s wise choice is to invest its entire R&D funds to get as much profit as possible. On the other hand, the firm’s marginal R&D investment is no lower than the marginal revenue at first and then lower than it. In other words, with increasing R&D investment the firm will lose money first and then make money. Therefore, if the R&D funds are sufficient to make the firm benefit, it invests all for as much profit as possible, otherwise it does not conduct R&D to avoid losses.

We then solve the NEV firm’s optimal R&D investment strategies when its marginal R&D investment is increasing, or $i_{qq}>0$. Let $i_{2}$ be the optimal R&D investment, and we can get proposition 2 as follows.

**Proposition 2**. In the case of $i_{qq}>0$:

(a) When the initial value of marginal R&D investment is lower than the marginal revenue, if the firm’s R&D funding is sufficient enough, the optimal R&D investment strategy is making the marginal R&D investment equal to the marginal revenue, otherwise the entire R&D funds;

(b) When the initial value of marginal R&D investment is no lower than the marginal revenue, the optimal R&D investment strategy is zero.

**Proof of Proposition 2**. Unlike the slope of the R&D investment curve in proposition 1, this has an increasing slope, that is, $i_{qq}>0$.

From Fig 3A, if $i_{q}\left|_{i=0}\right.<\bar{p}$, the firm obtains its unique maximum profits when $i_{q}\left|_{i=i_{1}}\right.=\bar{p}$, and the optimal R&D investment strategy is $i^{*}=i^{\max}$ if $i_{1}\geq i^{\max}$, otherwise, its optimal R&D investment strategy is $i^{*}=i_{1}$.

**Fig 3A. Changes in profits and revenue when** $i_{qq}>0$ **and** $i_{q}\left|_{i=0}\right.<\bar{p}$**.**

From Fig 3B, if $i_{q}\left|_{i=0}\right.\geq\bar{p}$, the slope of the R&D investment curve is always higher than marginal revenue, and the firm’s unique maximum profits at $i^{*}=0$.

**Fig 3B. Changes in profits and revenue when $i_{qq}>0$ and** $i_{q}\left|_{i=0}\right.\geq\bar{p}$**.**

In sum, the optimal R&D investment strategies with $i_{qq}>0$ are

$i^{\text{*}}=\left\{\begin{matrix} i^{\max}\text{ }i_{q}\left|_{i=0}\right.<\bar{p}\text{ }and\text{ }i_{1}\geq i^{\max} \\ i_{1}\text{ }i_{q}\left|_{i=0}\right.<\bar{p}\text{ }and\text{ }i_{1}<i^{\max} \\ 0\text{ }i_{q}\left|_{i=0}\right.\geq\bar{p} \end{matrix}\right.$.

Q.E.D.

Proposition 2 shows on one hand, when the firm’s marginal R&D investment is lower than the marginal revenue first and then higher than it, the firm can obtain maximum profits by making its marginal R&D investment equal to marginal revenue. However, the firm’s R&D funding is limited. If the firm can make its marginal R&D investment equal to marginal revenue, it will invest the required R&D funding, otherwise it will invest its entire R&D funding to get as much profit as possible. On the other hand, when the firm’s marginal R&D investment is no lower than the marginal revenue, the marginal R&D investment is always higher than marginal revenue, and the wise choice is not R&D.

**Crowding-in effect**

In this section, we solve the crowding-in effect of product subsidy by comparing the NEV firm’s optimal R&D investment strategies with and without the subsidy. The superscript “” of variables implies that the government doesn’t provide product subsidies.

First, referring to the previous section, we solve the firm’s optimal R&D investment strategies without product subsidy. It is easy to solve when there is $i_{qq}<0$, the optimal R&D investment strategies are

$i^{N*}=\left\{\begin{matrix} \text{0 }i_{q}\left|_{i=0}\right.>p\text{ }and\text{ }i_{2}^{N}>i^{\max} \\ i^{\max}\text{ }i_{q}\left|_{i=0}\right.>p\text{ }and\text{ }i_{2}^{N}\leq i^{\max}\text{ }or\text{ }i_{q}\left|_{i=0}\right.\leq p\text{ } \end{matrix}\right.$.

when there is $i_{qq}>0$, the optimal R&D investment strategies are

$i^{N*}=\left\{\begin{matrix} i^{\max}\text{ }i_{q}\left|_{i=0}\right.<p\text{ }and\text{ }i_{1}^{N}\geq i^{\max} \\ i_{1}^{N}\text{ }i_{q}\left|_{i=0}\right.<p\text{ }and\text{ }i_{1}^{N}<i^{\max}\text{ } \\ 0\text{ }i_{q}\left|_{i=0}\right.\geq p \end{matrix}\right.$;

Before comparing the optimal R&D investment strategies, two pairs of values for the optimal R&D investment, that is $i_{1}^{N}$and $i_{1}$, and $i_{2}^{N}$, and $i_{2}$ need to be compared.

**Lemma 2.** (a) When $i_{qq}<0$, there is $i_{1}<i_{1}^{N}$; (b) When $i_{qq}>0$, there is $i_{2}>i_{2}^{N}$.

**Proof of Lemma 2.** When $i_{qq}<0$, we know that $i_{1}^{N}$ satisfies $\pi^{N}\left|_{i^{N*}=i_{1}^{N}}\right.=pq\left(i_{1}^{N}\right)-i_{1}^{N}=pq_{0}$ and $i_{1}$ satisfies $\pi\left|_{i^{*}=i_{1}}\right.=\bar{p}q\left(i_{1}\right)-i_{1}=\bar{p}q_{0}$.

Then, $pq\left(i_{1}\right)-i_{1}=\pi_{0}=pq_{0}-s\left(q\left(i_{1}\right)-q_{0}\right)$. As $q\left(i_{1}\right)-q_{0}>0$, $pq\left(i_{1}\right)-i_{1}<pq\left(i_{1}^{N}\right)-i_{1}^{N}$.

Further, from $\pi^{N}=pq\left(i^{N}\right)-i^{N}$, $i_{q}>0$, and $i_{qq}<0$, if $i_{q}<p$, the bigger $\pi^{N}$, the bigger the corresponding $i^{N}$. As $i_{q}\left|_{i^{*}=i_{1}}\right.>\bar{p}$ and $i_{q}^{N}\left|_{i^{N*}=i_{1}^{N}}\right.>p$, we can get $i_{1}<i_{1}^{N}$.

When $i_{qq}>0$, we know that $i_{2}^{N}$ satisfies $i_{q}\left|_{i=i_{2}^{N}}\right.=p$ and $i_{2}$ satisfies $i_{q}\left|_{i=i_{2}^{N}}\right.=\bar{p}$. As $i_{q}\left|_{i=i_{2}^{N}}\right.=p<\bar{p}=i_{q}\left|_{i=i_{2}^{N}}\right.$ and $i_{qq}>0$, $i_{2}^{N}<i_{2}$.

Q.E.D.

Lemma 2 shows on the one hand, when the firm’s marginal R&D investment is decreasing, and its initial value of marginal R&D investment is higher than the marginal revenue without product subsides, the firm has an R&D investment making its profit equal to one conducting no R&D. As product subsidies will increase marginal revenue, which also leads to higher marginal profits, the firm can make profit conducting no R&D at a lower R&D investment than without product subsidy.

On the other hand, when the firm’s marginal R&D investment is increasing, and its initial value of marginal R&D investment is lower than marginal revenue without product subsidies, the firm has the optimal R&D investment to achieve its maximum profits. As product subsidy increases marginal revenue, the firm can make profits at a higher marginal R&D investment. In other words, only if the firm makes a higher R&D investment than without product subsidy, its marginal profits will become zero. This implies that it gets higher maximum profits at higher R&D investments.

We then solve the impact of product subsidy on the firm’s R&D investment when $i_{qq}<0$ and $i_{qq}>0$. Let $\Delta$ be the difference between the optimal R&D investment with and without product subsidy, and we can get propositions 3 and 4 as follows.

**Proposition 3**. In the case of $i_{qq}<0$:

(a) Only if the firm cannot benefit from R&D due to funds constraints, and the unit subsidy is large enough, it has a crowding-in effect;

(b) If there is a crowding-in effect, it always equals the firm’s entire R&D funds.

**Proof of Proposition 3**. When $i_{qq}<0$, (4) and (6) are compared. The results are shown in Table 1.

Q.E.D.

**Table 1. Crowding-in effect when $i_{qq}<0$.**

| R&D investment  without product subsidy | R&D investment with  product subsidy | The crowding-in effect |
| --- | --- | --- |
| $p<i_{q}\left\vert_{i=0}\right.\text{ }and\text{ }i^{\max}<i_{1}^{N}$ | $\bar{p}\leq i_{q}\left\vert_{i=0}\right.\text{ }and\text{ }i^{\max}<i_{1}<i_{1}^{N}$ | $\Delta=0$ |
| $p<i_{q}\left\vert_{i=0}\right.\text{ }and\text{ }i^{\max}<i_{1}^{N}$ | $\bar{p}\leq i_{q}\left\vert_{i=0}\right.\text{ }and\text{ }i_{1}\leq i^{\max}<i_{1}^{N}$ | $\Delta=i^{\max}$ |
| $p<i_{q}\left\vert_{i=0}\right.\text{ }and\text{ }i^{\max}<i_{1}^{N}$ | $\bar{p}>i_{q}\left\vert_{i=0}\right.$ | $\Delta=i^{\max}$ |
| $p<i_{q}\left\vert_{i=0}\right.\text{ }and\text{ }i_{1}^{N}\leq i^{\max}$ |  | $\Delta=0$ |
| $p\geq i_{q}\left\vert_{i=0}\right.$ |  | $\Delta=0$ |

Proposition 3 shows the crowding-in effect of product subsidy in cases where the NEV firm has a decreasing marginal R&D investment.

When the firm’s marginal revenue is lower than the initial value of the marginal R&D investment, its profits decrease first and then increase. This implies that there is an R&D investment threshold which makes the firm’s profits equal to conducting no R&D. If the firm’s R&D investment exceeds the threshold, the higher the firm’s R&D investment is the higher its profits are. As product subsidy increases the marginal revenue, it decreases the R&D investment threshold. Then, if the unit subsidy is large enough it will reduce the threshold to lower than the firm’s whole R&D funding, and the firm will invest all as a response. Otherwise, the firm doesn’t conduct R&D, because conducting R&D leads to losses even with product subsidies.

The other case is that if the firm only conducts R&D with product subsidies, it always invests all for maximum profit. This implies that the crowding-in effect will not increase with the unit subsidy.

**Proposition 4**. In the case of $i_{qq}>0$:

(a) If the firm cannot benefit from R&D without product subsidies, only if the unit subsidy is large enough, there is a crowding-in effect;

(b) If the firm has conducted R&D without product subsidies, there is always a crowding-in effect except it has no remaining R&D funds;

(c) If there is a crowding-in effect, it increases with the unit subsidy until the firm invests its entire R&D funds.

**Proof of Proposition 4**. When $i_{qq}>0$, (5) and (7) are compared. The results are shown in Table 2.

Q.E.D.

**Table 2. Crowding-in effect when $i_{qq}>0$.**

| R&D investment  without product subsidy | R&D investment with  product subsidy | The crowding-in effect |
| --- | --- | --- |
| $p\leq i_{q}\left\vert_{i=0}\right.$ | $\bar{p}\leq i_{q}\left\vert_{i=0}\right.$ | $\Delta=0$ |
| $p\leq i_{q}\left\vert_{i=0}\right.$ | $\bar{p}>i_{q}\left\vert_{i=0}\right.\text{ }and\text{ }i_{2}<i^{\max}$ | $\Delta=i_{2}$ |
| $p\leq i_{q}\left\vert_{i=0}\right.$ | $\bar{p}>i_{q}\left\vert_{i=0}\right.\text{ }and\text{ }i_{2}\geq i^{\max}$ | $\Delta=i^{\max}$ |
| $p>i_{q}\left\vert_{i=0}\right.\text{ }and\text{ }i_{2}^{N}<i^{\max}$ | $\bar{p}>i_{q}\left\vert_{i=0}\right.\text{ }and\text{ }i_{2}^{N}<i_{2}<i^{\max}$ | $\Delta=i_{2}-i_{2}^{N}$ |
| $p>i_{q}\left\vert_{i=0}\right.\text{ }and\text{ }i_{2}^{N}<i^{\max}$ | $\bar{p}>i_{q}\left\vert_{i=0}\right.\text{ }and\text{ }i_{2}^{N}<i^{\max}\leq i_{2}$ | $\Delta=i^{\max}-i_{2}^{N}$ |
| $p>i_{q}\left\vert_{i=0}\right.\text{ }and\text{ }i_{2}^{N}\geq i^{\max}$ | $\bar{p}>i_{q}\left\vert_{i=0}\right.\text{ }and\text{ }i^{\max}<i_{2}^{N}<i_{2}$ | $\Delta=0$ |

Proposition 4 shows the crowding-in effect of product subsidy in cases where the firm has an increasing marginal R&D investment.

On one hand, when the firm cannot benefit from R&D without product subsidy, it means that the marginal revenue is always lower than its marginal R&D investment. In other words, R&D leads to losses. Therefore, only if the product subsidy makes the marginal revenue higher than the initial value of marginal R&D investment, the firm can have positive marginal profits and profits. In contrast, product subsidy cannot produce a crowding-in effect.

On the other hand, if the firm can benefit from R&D without product subsidies, as a higher unit subsidy increases the firm’s marginal revenue, the firm is able to benefit at a higher marginal R&D investment. That is, the optimal R&D investment increases with the unit subsidy, as well as the crowding-in effect. However, as the firm has funding constraints, the highest crowding-in effect is the firm’s entire R&D funding. The results hold when the firm can benefit from R&D without product subsidies.

**Numerical analyses**

In this section, we use choose an arbitrary quantity test our model. Let $a\left(i\right)=e^{vi}$, where $v$ is the firm’s R&D capability coefficient and $a_{0}=a\left(0\right)=1$. Readily shown, $i_{q}>0$ and $i_{qq}<0$, which indicates that the firm has a decreasing marginal R&D investment. Moreover, the marginal R&D investment function is $i_{q}=a_{i}^{-1}=v^{-1}e^{-vi}$, where $i_{q}\left|_{i=0}\right.=v^{-1}$. The firm’s profits function is $\pi\left(i\right)=\left(p+s\right)e^{vi}-i$.

Where $i^{\max}=1.9$, $x=0.5$ and $p=0.5$, we can calculate the initial value of marginal R&D investment as $i_{q}\left|_{i=0}\right.=2$. From proposition 1, we know that $i^{N*}=0$. Thereafter, we show that the changes of the crowding-in effect of the unit subsidy increases from $0$ to $1$.

In Fig 4, one case shows that the NEV firm has a decreasing marginal R&D investment, and its initial value of marginal R&D investment is higher than the marginal revenue. More specifically, establish a unit subsidy threshold, whereby $s=0.32$, which, in turn, determines whether the crowding-in effect is present. Firstly, in this case a unit subsidy greater than $0.32$, shows that the crowding-in effect is present. Secondly, a unit subsidy greater than $0.32$indicates that the firm ordinarily invests its R&D funds in their entirety.

**Fig 4. The crowding-in effect when $i_{qq}<0$.**

We then take into consideration that the NEV firm has an increasing marginal R&D investment. Let $a\left(i\right)=\bar{x}-e^{-vi}$, where $\bar{x}$ is the quality of ICEVs and $a_{0}=\bar{x}-1$. Readily shown, $i_{q}>0$ and $i_{qq}>0$. Moreover, the marginal R&D investment function is $i_{q}=a_{i}^{-1}=v^{-1}e^{vi}$, where $i_{q}\left|_{i=0}\right.=v^{-1}$. The firm’s profits function is $\pi\left(i\right)=\left(p+s\right)\left(\bar{x}-e^{-vi}\right)-i$.

Let $i^{\max}=1.9$, $v=0.5$, and $p=1$ or $p=3$. Easy to get $i_{q}\left|_{i=0}\right.=2$. From proposition 2, we know that $i^{N*}=0$ or $i^{N*}=0.81$. Then, we show the changes in the crowding-in effect when the unit subsidy increases from $0$ to $5$.

In Fig 5A, the NEV firm has an increasing marginal R&D investment. The initial value of marginal R&D investment of the firm is higher than the marginal revenue, that is, $i_{q}\left|_{i=0}\right.=2>1=p$. We can see that the crowding-in effect doesn’t exist when the unit subsidy is lower than $1$. After the unit subsidy exceeds $1$, the crowding-in effect increases with it from $1$ to $4.17$ first, and then equals $i^{\max}=1.9$. This means that only if the unit subsidy is large enough, which should be higher than $1$ in this case, the product subsidy has a crowding-in effect. However, if the unit subsidy is higher than $4.17$, the crowding-in effect will remain unchanged. The reason for this is that the firm will ordinarily invest its entire R&D budget subject to financial constraints.

**Fig 5A. Crowding-in effect when $i_{qq}>0$ and $p=1$.**

In Fig 5B, shows that the NEV firm can benefit from R&D without a product subsidy, and the crowding-in effect will increase as the unit subsidy increases from $0$ to $2.17$ until the firm invests its whole available R&D budget.

**Fig 5B. Crowding-in effect when $i_{qq}>0$ and $p=3$.**

**Conclusions**

Given that the early adopter group’s consumers have an increasing density on acceptable quality thresholds, we proposed a vertical quality differentiation model of product R&D with subsidy. The impact of product subsidies on the NEV firm’s R&D investment is discussed. We hope to have shed some light on how to deploy and improve government product subsidy policies rationally.

The NEV firms’ marginal R&D investment supported by increases in sales may fluctuate according to the increasing density in early adopters with minimum acceptable quality thresholds. We show that firms with a decreasing marginal R&D investment are either reluctant to undertake R&D, or unable to increase their investment in R&D due to budget constraints. At best, firms that are unable to conduct their own R&D can benefit from the granting of significant unit product subsidies, whereby these produce a crowding-in affect equivalent to their entire R&D budgets. By contrast, firms that are not constrained by marginal increases in R&D investment, inevitably face the dilemma of lower profits against increases in R&D investments. In general, the crowding-in effect from product subsidies result in higher company profit margins. More specifically, firms who rely on subsidies to conduct R&D will only do so with adequate unit grants. Thus, increases in unit subsidies and the crowding-in effect are interdependent. However, the aforementioned conclusions are limited to those firms that are financially constrained by having spent their entire R&D budget, and are unable to make use of subsidies to produce or increase a crowding-in effect.

Furthermore, governments are advised to consider the quality preferences of early adopter groups. Based on a situational analysis, subsidies should be granted to NEV firms, provided there is a both a decrease in their marginal R&D investment, and they rely on subsidies to conduct R&D. The appropriate amount of the unit subsidies should be calculated accordingly at the equivalent baseline formation of a crowding-in effect. The reasonable alternative hereto is to replace the product subsidy policy with a funding support policy. In contrast, if firms increase their marginal R&D investment, it becomes necessary to achieve or improve the crowding-in effect by adjusting the unit subsidy. However, if firms are financially constrained by having spent their entire R&D budgets, the formation of a greater crowding-in effect requires facilitation through a funding support policy.

**Acknowledgements**

This research is supported by the National Natural Science Foundation of China (No. 71573025), Chinese National Funding of Social Sciences (No. 17XGL008), and the Fundamental Research Funding for the Central Universities (No. 2018CDXYJG0040).

**References**

1. Li W, Long R, Chen H. Consumers’ evaluation of national new energy vehicle policy in China: An analysis based on a four paradigm model. 2016;99:33-41. doi: [10.1016/j.enpol.2016.09.050](http://dx.doi.org/10.1016/j.enpol.2016.09.050)
2. Zhang L, Qin Q. China’s new energy vehicle policies: evolution, comparison and recommendation. TRANSPORT RES A. 2018;110:57-72. [doi: 10.1016/j.tra.2018.02.012](https://doi.org/10.1016/j.tra.2018.02.012)
3. Jensen AF, Mabit SL. The use of electric vehicles: A case study on adding an electric car to a household. TRANSPORT RES A. 2017;106:89-99. [doi: 10.1016/j.tra.2017.09.004](http://dx.doi.org/10.1016/j.tra.2017.09.004)
4. Jing W, Ramezani M, An K, Kim I. Congestion patterns of electric vehicles with limited battery capacity. PLOS ONE. 2018;13(3):e0194354. [doi: 10.1371/journal.pone.0194354](https://doi.org/10.1371/journal.pone.0194354)
5. Rahmani D, Loureiro ML. Why is the market for hybrid electric vehicles (HEVs) moving slowly? PLOS ONE. 2018;13(3):e0193777. [doi: 10.1371/journal.pone.0193777](https://doi.org/10.1371/journal.pone.0193777)
6. Bjerkan KY, Nørbech TE, Nordtømme ME. Incentives for promoting Battery Electric Vehicle (BEV) adoption in Norway. TRANSPORT RES D. 2016;43:169-180. [doi: 10.1016/j.trd.2015.12.002](http://dx.doi.org/10.1016/j.trd.2015.12.002)
7. Shafieia E, Davidsdottira B, Fazelia R, Leaverb J, Stefanssonc H, Asgeirsson EI. Macroeconomic effects of fiscal incentives to promote electric vehicles in Iceland: Implications for government and consumer costs. ENERG POLICY. 2018;114:431-443. doi: 10.1016/j.enpol.2017.12.034
8. Li W, Long R, Chen H, Yang T, Geng J, Yang M. Effects of personal carbon trading on the decision to adopt battery electric vehicles: Analysis based on a choice experiment in Jiangsu, China. APPL ENERG. 2018;209;478-488. doi: 10.1016/j.apenergy.2017.10.119
9. Wang N, Tang L, Pan H. Effectiveness of policy incentives on electric vehicle acceptance in China: A discrete choice analysis. TRANSPORT RES A. 2017;105:210-218. doi: 10.1016/j.tra.2017.08.009
10. Li W, Long R, Chen H, Yang M, Chen F, Zheng X, et al. Would personal carbon trading enhance individual adopting intention of battery electric vehicles more effectively than a carbon tax? RESOUR CONSERV RECY. 2019;149;638-645. doi: 10.1016/j.resconrec.2019.06.035
11. Ministry of Science and Technology of the People’s Republic of China. Notice on Carrying Out the Pilot Work of Demonstration and Promotion of Energy Conservation and New Energy Vehicles. MOST homepage. 23 Jun 2009 [Cited 2020 March 14]. Available from: <http://www.most.gov.cn/fggw/zfwj/zfwj2009/200902/t20090224_67588.htm>
12. Souhu auto. new energy vehicle subsidy Announcement: cumulatively exceeded 100 billion Chinese yuan. Souhu homepage. 28 Mar 2018 [Cited 2020 March 14]. Available from: <https://www.sohu.com/a/233217181_100113582>
13. Zhang L, Qin Q. China’s new energy vehicle policies: evolution, comparison and recommendation. TRANSPORT RES A. 2018;110:57-72. [doi: 10.1016/j.tra.2018.02.012](https://doi.org/10.1016/j.tra.2018.02.012)
14. Liu Y, Kokko A. Who does what in China’s new energy vehicle industry? ENERG POLICY. 2013;57:21-29. doi: 10.1016/j.enpol.2012.05.046
15. Lou JW. Improve fiscal policies and innovate institutional mechanisms. China Financial and Economics News. 28 Jun 2016 [Cited 2020 March 14]. Available from: <http://www.cfen.com.cn/dzb/dzb/history/20160126/>
16. Arrow KJ. Economic Welfare and the Allocation of Resources for Invention. In: Universities-National Bureau Committee for Economic Research, Committee on Economic Growth of the Social Science Research Council, editors. The Rate and Direction of Inventive Activity: Economic and Social Factors. Princeton University Press. 1962:609-626.
17. Spence M. Cost Reduction, Competition, and Industry Performance. ECONOMETRICA. 1984;52(1):101-122.
18. Liu D, Chen T, Liu X, Yu Y. Do more subsidies promote greater innovation? Evidence from the Chinese electronic manufacturing industry. ECON MODEL. 2019;80:441–452. doi: 10.1016/j.econmod.2018.11.027
19. Huergo E, Moreno L. Subsidies or loans? Evaluating the impact of R & D support programmes. RES POLICY. 2017;46:1198–1214. doi: 10.1016/j.respol.2017.05.006
20. Lin B, Luan R. Do government subsidies promote efficiency in technological innovation of China’s photovoltaic enterprises? J CLEAN PROD. 2020;254:120108. doi: 10.1016/j.jclepro.2020.120108
21. Meuleman M, Maeseneire WD. Do R&D subsidies affect SMEs’ access to external financing? RES POLICY. 2012;41:580-592. doi: 10.1016/j.respol.2012.01.001
22. Catozzella A, Vivarelli M. The possible adverse impact of innovation subsidies: some evidence from Italy. Int Entrep Manag J. 2016;12:351–368. doi: 10.1007/s11365-014-0342-3
23. Gorg H, Strobl E. The Effect of R&D Subsidies on Private R&D. Economica. 2007;74:215-234. doi: 10.1111/j.1468-0335.2006.00547.x
24. Montmartin B, Herrera M. Internal and external effects of R&D subsidies and fiscal incentives: Empirical evidence using spatial dynamic panel models. RES POLICY. 2015;44:1065-1079. doi: 10.1016/j.respol.2014.11.013
25. David PA, Hall BH. Heart of darkness: modeling public–private funding interactions inside the R&D black box. RES POLICY. 2000,29:1165-1183. doi: 10.1016/s0048-7333(00)00085-8
26. Hassinea HB, Mathieu C. R&D crowding out or R&D leverage eﬀects: An evaluation of the French cluster-oriented technology policy. TECHNOL FORECAST SOC. 2020;155:120025. doi: 10.1016/j.techfore.2020.120025
27. Bai Y, Song S, Jiao J, Yang R. The impacts of government R&D subsidies on green innovation: evidence from Chinese energy-intensive firms. J CLEAN PROD. 2019;233:819-829. doi: 10.1016/j.jclepro.2019.06.107
28. Yu F, Guo Y, Le K, Barnes JS, Zhang W. The impact of government subsidies and enterprises’ R&D investment. ENERG POLICY. 2016;89:106-113. doi: 10.1016/j.enpol.2015.11.009
29. Yang D, Xiao T. Pricing and green level decisions of a green supply chain with governmental interventions under fuzzy uncertainties. J CLEAN PROD. 2017;149:1174-1187. doi: 10.1016/j.jclepro.2017.02.138
30. Howell ST. Financing Innovation: evidence from R&D Grants. AM ECON REV. 2017;107(4):1136-1164. doi: 10.1257/aer.20150808
31. Jiang C, Zhang Y, Bu M, Liu W. The Effectiveness of Government Subsidies on Manufacturing Innovation: evidence from the New Energy Vehicle Industry in China. SUSTAIN. 2018;10:1692. doi: 10.3390/su10061692
32. Bergek A, Berggren C, KITE Research Group. The impact of environmental policy instruments on innovation: A review of energy and automotive industry studies. ECOL ECON. 2014;106:112-123. doi: 10.1016/j.ecolecon.2014.07.016
33. Albrecht AEG. Policy Instruments and Incentives for Environmental R&D:A Market-Driven Approach. SSRN ELECTRON J. 1999. doi: 10.2139/ssrn.158818
34. Xiong Y, Fan S, Liu X. The Difference of Fiscal Subsidy for New Energy Vehicles and R&D Investment intensity of the Manufacturers: An Analysis Based on the Heterogeneity of the Manufacturer’s Strategic Decision. SCI SCI MANGE S T. 2018;39(6);72-83. doi: CNKI:SUN:KXXG.0.2018-06-007
35. Liu L, Zhao Z. Simulation of the running mechanism of multi-agent innovation network when fiscal subsidy drops out: A case of new energy vehicles. SCI RES MANAG. 2016;37(8);58-66. doi: 10.19571/j.cnki.1000-2995.2016.08.007
36. Rogers EM. Attributes of Innovations and Their Rate of Adoption. In: Rogers EM, editors. Diffusion of innovations. A Division of Simon & Schuster: New York; 2003. pp. 115-153.
37. Moore GA. Crossing the Chasm: Marketing and Selling High-Tech Products to Mainstream Consumers. In: Moore GA, editors. Harper business, New York. 2014.
38. Hardman S, Tal G. Who are the early adopters of fuel cell vehicles? INT J HYDROGEN ENERG. 2018;43:17857-17866. doi: 10.1016/j.ijhydene.2018.08.006
39. Cecere C, Corrocher N, Guerzoni M. Price or performance? A probabilistic choice analysis of the intention to buy electric vehicles in European countries. ENERG POLICY. 2018;118:19-32. doi: 10.1016/j.enpol.2018.03.034
40. Chu W, Im M, Song YM, Park J. Psychological and behavioral factors aﬀecting electric vehicle adoption and satisfaction: A comparative study of early adopters in China and Korea. TRANSPORT RES D. 2019;76:1-18. [doi: 10.1016/j.trd.2019.09.009](https://doi.org/10.1016/j.tra.2018.02.012)
41. Souhu auto. The number of new energy vehicles in our country was 3.44 million until June 2019. Souhu homepage. 5 Jul 2019 [Cited 2020 March 14]. Available from: https://www.sohu.com/a/325120675_733088
42. Santos G, Davies H. Incentives for quick penetration of electric vehicles in five European countries: Perceptions from experts and stakeholders. TRANSPORT RES A. Forthcoming. doi: 10.1016/j.tra.2018.10.034
43. Li W, Long R, Chen H, Geng J. A review of factors influencing consumer intentions to adopt battery electric vehicles. RENEW SUST ENERG RNEV. 2017;78; 318-328. doi: 10.1016/j.rser.2017.04.076
44. Jensen FA, Mabit LS. The use of electric vehicles: A case study on adding an electric car to a household. TRANSPORT RES A. 2017;106:89-99. [doi: 10.1016/j.tra.2017.09.004](https://doi.org/10.1016/j.tra.2018.02.012)
45. Li W, Long R, Chen H, Chen F, Zheng X, He Z, et al. Willingness to pay for hydrogen fuel cell electric vehicles in China: A choice experiment analysis. INT J HYDROGEN ENERG. Forthcoming. doi: 10.1016/j.ijhydene.2020.01.046
46. Li W, Long R, Chen H, Dou B, Chen F, Zheng X, et al. Public Preference for Electric Vehicle Incentive Policies in China: A Conjoint Analysis. INT J ENVIRON RES PUBLIC HEALTH. 2020;17:318. doi:10.3390/ijerph17010318
47. Kwona Y, Sonb S, Jang K. Evaluation of incentive policies for electric vehicles: An experimental study on Jeju Island. 2018;116:404-412. doi: 10.1016/j.tra.2018.06.015
48. Hackbarth A, Madlener R. Willingness-to-pay for alternative fuel vehicle characteristics: A stated choice study for Germany. TRANSPORT RES A. 2016;85:89-111. [doi: 10.1016/j.tra.2015.12.005](https://doi.org/10.1016/j.tra.2018.02.012)
49. Neaimeh M, Salisbury DS, Hill AG, Blythe TP, Scoffield RD, Francfort EJ. Analysing the usage and evidencing the importance of fast chargers for the adoption of battery electric vehicles. ENERG POLICY. 2017;108:474-486. doi: 10.1016/j.enpol.2017.06.033
50. Noel L, Rubens ZG, Kester J, Sovacool KB. Understanding the socio-technical nexus of Nordic electric vehicle (EV) barriers: A qualitative discussion of range, price, charging and knowledge. ENERG POLICY. 2020;138:111292. doi: 10.1016/j.enpol.2020.111292
51. Mussa M, Rossen S. Monopoly and product quality. J ECON THEORY. 1978;18:301-317.
52. Sällström S. Technological Progress and the Chamberlin Effect. 2003;47(4):427-449. doi: 10.1111/1467-6451.00107
53. Saha S. Firm’s objective function and product and process R&D. ECON MODEL. 2014;36:484-494. doi: 10.1016/j.econmod.2013.08.041
